# Annotations

**Published:** 1898-02-05

**Authors:** 


### ANNOTATIONS.

### The Röntgen Rays.

Two articles appear this month on the Röntgen rays, one in the *American Journal of the Medical Sciences* by Dr. William White, the other in the *Humanitarian* by Dr. David Walsh, both of them very interesting and suggestive, both showing how great have been the advances made during the short time which has elapsed since the discovery of the process, and both showing also how much there is yet to learn. We still are dealing with a medium of the real nature of which we know next to nothing, and even our processes are mostly empirical. Yet we do know enough to hint that with greater knowledge is likely to come far greater usefulness. The fact mentioned by Dr. Walsh, that sometimes, apparently by mere accident, rays are obtained by which structures can be differentiated, points to the possibility of sometime displaying far more distinctly than at present the various tissues of the body. Dr. White summarises a few of the attempts to obtain therapeutic help from the rays in the treatment of disease. What is certain is that in passing through various structures the rays are not entirely idle, and the well-known trophic changes which are produced in the skin, the hair, the nails, and other parts, are sufficient to assure us that under some conditions these rays in their passage through the body are capable of exerting or setting free considerable energy, and also of exercising great power of selection, so that again we see that a little increase of knowledge may enable us to select rays according to the tissues we wish to treat, as well as according to those which we desire to depict. These are dreams no doubt—to-day. But to-morrow they may be actualities.

### Brain Fatigue in School Work.

A QUESTION of considerable interest to those who have the management of elementary schools is raised by a paper by Dr. Kemsies in the *Deut. Med. Wochenschrift.* Dr. Kemsies is the head master of a large German school, and he gives his personal experience as to the conditions which influence the working capacity of his pupils. The best work, he says, is done at the beginning of the week, after the Sunday holiday; and by Tuesday afternoon it has already begun to deteriorate. Again, the mornings produce the best work, and the mid-day rest, during which the mid-day meal is taken, does not produce the same recuperation as the night's rest. If these results are to be taken as correct it would seem as if many of our educational customs might be reformed with considerable advantage. We have long thought that a reversion to the two half-holidays would be a great advantage to the children, however much the teachers might dislike it, and these investigations only tend to confirm our idea. Young ladies, again, used to go to school in the morning and the afternoon, with a two hours' interval between the two sessions. But now it is thought desirable, we suppose, that they should be free to pay calls with their mothers in the afternoons, and everything is crowded into one long grind of four hours in the morning. Moreover, a modern blackboard lesson is a very different thing from the work that used to be done in school hours, much of which would now be called preparation; and, although as a means of teaching facts its value is obvious, so also is its power of producing fatigue. Curiously enough, the German experience is that gymastics, which we are apt to class with play, produce the greatest fatigue of all, rendering the work done after it practically useless. But, then, the gymnastics are probably done in a class, each pupil having to do as he is told. This is practically another lesson, and is not to be put into the same category with half-an-hour in a fives-court, or at football. It must not be forgotten that the effort to make teaching interesting, which is its great characteristic in modern times, does not really lighten the burden on the child. It makes learning easier, but it makes him learn more; it keeps him always at it, and it steals from him those moments of torpor and stupidity, of dreams and vacancy, in which his little brain used to take furtive snatches of repose.

### The Workhouse Ambulance.

BY degrees the workhouse infirmaries are being transformed into great hospitals for the treatment of acute disease. It is not only natural, but it is quite right, that with such well-equipped institutions ready for the reception of the sick, the district medical officers should transfer to them their more serious cases of illness. Yet, although this is the natural and proper result of the improvements which have taken place in the construction and management of our workhouse infirmaries, the guardians seem sadly backward in recognising the responsibilities which attach to them in consequence of the changed condition of affairs. If the infirmaries are to be supplied with resident medical officers, with nurses, and with all the appliances of hospitals for the treatment of acute disease, and if ambulances are provided by which patients may be brought from their homes, surely it is not too much to ask that some sort of care shall be taken of these unfortunate people while hey are being carried from place to place. The evidence, however, which was given at an inquest held last week on a woman who died in an ambulance while being transferred from her home in Kensal New Town to the Chelsea Infirmary tends to show that such is not the case. No nurse was provided, no light, no stimulants; so the woman simply died. The medical evidence was to the effect that with proper attendance and stimulants her life might have been prolonged. The relieving officer stated that he had no power to provide a nurse; and the parish medical officer said that, although it was most advisable that a nurse should be in attendance at such removals, there was no way of getting one; and on the coroner saying, "If I were in your place I should refuse to sign an order for removal unless a nurse were provided," the medical officer could but answer, "My regulations do not allow me to refuse." And so it is. While an outdoor pauper the patient is under one administration, when an indoor pauper under another, and between the two there is a gap bridged over by an ambulance and nothing else; in some country places only a cart. The guardians seem to forget that if they so arrange their outdoor relief that severe cases of illness must be removed, they are responsible for their safe removal.

